# Effects of dietary N-carbamylglutamate on rumen fermentation parameters, and bacterial community diversity of Holstein dairy cows in Tibet

**DOI:** 10.3389/fmicb.2023.1101620

**Published:** 2023-05-09

**Authors:** Jinjia Zhu, Yicheng Wu, Aoyu Jiang, Bin Li, Tao Ran, Chuanshe Zhou, Dingfu Xiao, Zhiliang Tan

**Affiliations:** ^1^CAS Key Laboratory for Agro-Ecological Processes in Subtropical Region, National Engineering Laboratory for Pollution Control and Waste Utilization in Livestock and Poultry Production, Hunan Provincial Key Laboratory of Animal Nutrition Physiology and Metabolic Process, Institute of Subtropical Agriculture, Chinese Academy of Sciences, Changsha, Hunan, China; ^2^College of Advanced Agricultural, University of the Chinese Academy of Sciences, Beijing, China; ^3^Institute of Animal Husbandry and Veterinary, Tibet Autonomous Regional Academy of Agricultural Sciences, Lhasa, Tibet, China; ^4^College of Pastoral Agriculture Science and Technology, Lanzhou University, Lanzhou, China; ^5^College of Animal Science and Technology, Hunan Agricultural University, Changsha, Hunan, China

**Keywords:** N-carbamylglutamate, rumen bacteria, rumen fermentation, Tibet, Holstein dairy cow

## Abstract

**Introduction:**

The Tibetan Plateau is characterized by low temperature and hypoxia. N-carbamylglutamic acid (NCG) can increase blood oxygen saturation, and have the potential to be used to prevent the high-altitude hypoxia stress state of cows. However, its beneficial effect on the rumen microbiota of Holstein dairy cows remains unclear.

**Methods:**

Hence, the experiments 12 multiparous (parity ranged from 2 to 7) Holstein dairy cows (413.0 ± 42 kg) were randomly assigned to 2 treatments with 6 replicates in each treatment: basal diet (CON, control group) and basal diet plus 20 g/d/cow of NCG (NCG, experiment group), respectively. To study the effects of dietary NCG supplementation on rumen microbiota of Holstein dairy cows in Tibet. The experiment lasted for 45 days, with 15 days of pre-feeding and 30 days of formal trail period.

**Results:**

The results showed that ruminal NH_3_-N concentration in NCG group was lower (*p* < 0.05) than that in the CON group, while molar proportion of acetic acid and total volatile fatty acid (VFA) concentration were increased (*p* < 0.05) with the addition of NCG. Microbial diversity increased (*p* < 0.05) in NCG group, with *Bacteroidetes*, *Firmicutes,* and *Patescibacteria* as the most abundant phyla. The KEGG pathway analysis showed that the potential function of ruminal bacteria was mainly enriched in metabolism (carbohydrates, amino acids, lipids, energy, and nucleotides) and genetic information processing (replication, repair, and translation).

**Conclusion:**

In conclusion, NCG can improve rumen nitrogen utilization, total VFA and acetic acid production, and increase rumen microbial diversity, all of which could make the introduced Holstein dairy cows to better adapt to the harsh environment in Tibet and improve their production performance.

## 1. Introduction

Due to the special geographical and environmental conditions, the Qinghai-Tibet Plateau has a long cold climate, strong ultraviolet radiation, strong wind and low oxygen content in the air (Liu H. et al., 2022; Liu Z. et al., 2022), leading to an extremely harsh living environment for livestock. As the hinterland of plateau, the living environment is even worse in Tibet, making it a major restricting factor for the development of Tibet’s animal husbandry. Local animals like Yaks (*Bos grunniens*) and Tibetan sheep are well-adapted to the harsh living environment in Tibet; however, introduced animals like Holstein dairy cows and Jersey cows could not stand with the harsh environment there. For example, introduced Jersey cows in this area have to live and produce under the challenge of low temperature and hypoxia, which is the major reason of hypoxemia and tissue hypoxia (Kong et al., 2019). Moreover, it is prone to a series of pathophysiological changes that will greatly affect the body’s health and production performance, such as pulmonary hypertension, decreased milk production, and other metabolic disorders and clinical symptoms (Neary and Garry, 2014).

Offering nutritional feed additives to animals that have the risk of suffering hypoxic have been proved efficient (Wenk, 2000). Although Arg (Arginine) could attenuate inflammatory stress and significantly improve lactating performance of dairy cows (Wang, 2016; Zhao et al., 2018; Ding et al., 2019), and improve the altitude reaction in high altitude animals by increasing blood oxygen saturation (Schneider et al., 2001), the rapid degradation in the rumen (Chacher et al., 2012) and the high price hindered its utilization in ruminant production. In the meanwhile, N-carbamoyl glutamate (NCG), a structural analog of N-acetylglutamate acts as an allosteric activator of essential cofactor aminophosphate synthase in endogenous arginine synthesis (Gessler et al., 2010), is much more stable than Arg in the rumen (Ma et al., 2019). The NCG is non-toxic to livestock (Guffon et al., 1995), and previous studies consistently proven that supplementation of NCG to ruminant diets could improve milk yield and production performance for dairy cows (Chacher et al., 2014; Ma et al., 2020; Gu et al., 2021), and improve reproduction of gestation Hu sheep (Sun et al., 2017) and improved the health and growth of suckling Hu lambs (Zhang et al., 2018). Therefore, it is widely used as a viable and cost-effective alternative to Arg. Our parallel study indicated that dietary supplementation of NCG by 20 g/d/head prevented hypoxic stress and tended to improve milk quality of Jersey dairy cows in Tibet (Liu H. et al., 2022; Liu Z. et al., 2022). This suggests that NCG also has beneficial effects on the physiological regulation of introduced animals at high altitude plateau.

This led to the hypothesis that supplementation of NCG would alter rumen metabolism via regulating ruminal microbial community of Holstein dairy cows that introduced to Tibet. Hence, this study was designed to assess the effect of NCG supplementation on rumen metabolism and microbiota of Holstein dairy cows that have been introduced to Tibet for a long time.

## 2. Materials and methods

### 2.1. Ethics statement

The animals and experimental procedures used in this study were approved by the Institutional Animal Care and the Use Committee, Institute of Subtropical Agriculture, Chinese Academy of Sciences, Changsha, PR China (Approval number: ISA-2019-0115).

### 2.2. Animals, experimental design, and diets

This study was conducted during the warn season (from June to July 2021) in Changzhu dairy farm (Latitude N: 28°57′17″, Longitude E: 91°39′4″, Altitude: 3700 m), Shannan City, Tibet Autonomous Region, China. The hybrid Holstein breed (Holstein × yellow cattle) was selected, Average milk yield 9.6 kg/d, dry matter intake 16.91 kg/d. Twelve multiparous (parity ranged from 2 to 7) Holstein dairy cows (413.0 ± 42 kg) in pre-lactation with no clinical signs of disease were used as experimental animals in a completely random designed experiment.

All Holstein dairy cows randomly assigned into two groups (6 cows/group). Both groups of cows were randomly assigned to one of two treatments, with one group fed a basal diet (CON group) and another group fed the basal diet plus 20 g NCG per day for each cow (NCG group). The basal diet was offered in the form of total mixed ration (TMR), while the NCG was offered by top-dressing on the basal TMR at morning feeding to make sure been fully consumed. The NCG, with an ≥80%, used in the current study was supplied by Nenghe Biotechnology Co., Ltd., Zhongshan, China. The ingredients and chemical compositions of the basal diet are illustrated in Table 1. The experiment lasted 45 days, with 15 days of pre-feeding and 30 days of formal experimentation. All cows were fed two equal meals at 06:00 and 18:00 h every day. All animals were allowed free access to feed and fresh water throughout the experimental period.

**Table 1:** Ingredients and nutrient compositions of the basal diet (DM basis).

Items	Content (%)
Ingredients	
Corn silage	62.50
Wheat grass	20.83
Alfalfa hay	8.33
Oat	0.92
Wheat bran	0.75
DDGS	0.67
Corn	4.37
Soybean meal	0.50
Cottonseed meal	0.42
Premix^1^	0.63
NaCl	0.08
Total	100
Nutrient levels^2^	
CP	9.23
EE	4.84
NDF	51.99
ADF	24.35
NE_L_/(MJ/kg)	5.15
Calcium	0.55
Phosphorus	0.26

^1^Per kilogram of premix contained the following: Ferric, 4,500 mg; Copper, 1,600 mg; Manganese, 3,000 mg; Zinc, 5,500 mg; Selenium, 30 mg; Cobalt, 20 mg; Iodine, 30 mg; vitamin A 600,000 IU; vitamin D 200,000 IU; vitamin E 200 IU.

^2^CP, crude protein; EE, ether extract; NDF, neutral detergent fiber; ADF, acid detergent fiber, NE_L_, milk production and net energy.

### 2.3. Sample collection and measurements

Diet offered and refused for each cow were recorded, and samples of diet and orts were collected daily throughout the formal experimental period. Feed and orts samples were dried at 65°C for 72 h, and ground to pass a 1 mm screen. Then the samples were analyzed for analytical dry matter (DM; method 934.01), ether extract (EE; method 920.39), crude protein (CP; method 976.06), acid detergent fiber (ADF, method 985.29), calcium (Ca; method 978.02), and total phosphorus (TP; method 946.06) according to the procedures of the Association of Official Analytical Chemist (AOAC, 2012). The neutral detergent fiber (NDF) contents were determined using a Fibretherm Fiber Analyzer with F57 filter bags (ANKOM A200, ANKOM Technology Corp., Fairport, NY, United States) according to Van Soest et al. (1991).

Rumen fluid samples (100 mL) were collected from each cow using an oral stomach tube at 2 h after morning feeding on the final day of the experiment as reported previously (Shen et al., 2012). The initial 50 mL was discarded to minimize contamination of oral saliva. The pH value was measured immediately using a pH meter (PHS-100 portable acidity meter, Tianqi Mdt InfoTech Ltd., Shanghai, China). Thereafter, rumen fluid samples were filtered through a 4-layer cheesecloth and stored at −80°C until analysis. During sample analysis, 10 mL of rumen fluid was used for microbial crude protein (MCP) determination, and 5 mL of rumen fluid was centrifuged (10,000 g, 4°C, 15 min) to obtain supernatants, which was used for volatile fatty acid (VFA) and ammonia nitrogen (NH_3_-N) determination. The VFA concentration was measured using high-performance liquid chromatography (Agilent 1,100, Santa Clara, CA), referring to the method of Wang et al. (2020). The treated sample was automatically injected into the Agilent DBFFAP gas capillary column (30 m × 0.25 mm × 0.25 μm). Injector temperature was set to 250°C and detector temperature was set to 280°C. The split ratio of all samples was set at 50:1. The column temperature was programmed to heat from 60 to 220°C at a rate of 20°C/min, and then held for 5 min. A colorimetric method was used to detect NH_3_-N and MCP as described by Wang et al. (2019).

### 2.4. DNA extraction, PCR amplification, and 16S rRNA sequencing

Total microbial DNA was extracted using the QIAamp^®^ DNA Stool Mini Kit (Qiagen, Hilden, Germany), and the quality and quantity of extracted DNA were measured by a NanoDrop 2000 (Thermo Scientific, Waltham, United States). The quantitative real-time PCR analysis of total bacteria was carried out with an ABI 7900 sequence detection system (Applied Biological System, Foster, CA, United States), using SYBR green premix Pro Taq HS qPCR kit AG11701 (Magigene Bioinformatics Technology, Guangzhou, China). The specific test procedures were in accordance with Jiao et al. (2015). The V3-V4 hypervariable region of 16S rRNA genes of bacteria was amplified with primer 338F (5′-ACT CCT ACG GGA GGC AGC A-3′) and 806R (5′-GGA CTA CHV GGG TWT CTA AT-3′) as reported by Mumbi et al. (2015). The PCR procedures and conditions were performed according to the method described by Jiao et al. (2016) The PCR products were purified using the QIAquick Gel Extraction Kit (QIAGEN, Germany), quantified using real-time PCR, and sequenced on the MiSeq platform at Magigene Bioinformatics Technology (Guangzhou, China). The raw paired-end reads analysis and the quality control were referenced to the methods as reported by Huang et al. (2020). Qualified reads were separated using sample-specific barcode sequences and trimmed using the Illumina Analysis Pipeline version 2.6. The dataset was then analyzed using QIIME 2. The sequences with a similarity level of more than 97% were clustered into the same operational taxonomic units (OTUs), using the method of Uparse Software (7.1 version).^1^ Analyses of alpha diversities were performed using the ggplot2 and vegan packages of R 1.7 version (Oksanen et al., 2013). All computed distance matrix was visualized in a non-metric multidimensional scaling (NMDS) graph. The R software was used to analyze the differences of beta diversity index between groups.

### 2.5. Correlation analysis of ruminal fermentation and rumen bacteria

Spearman correlation analysis was carried out to analyze the relationship between the relative abundances of rumen bacteria and ruminal fermentation parameters. The absolute value of correlation coefficient was greater than 0.6 and the *p* < 0.05, the correlation was considered significant. The R Pretty Heatmaps package was applied to obtain heatmaps to visualize the robustness of correlations.

### 2.6. Functional prediction of microbial pathway abundances by PICRUSt

The functional potential of rumen microbes was inferred from the ASVs table using PICRUSt (Phylogenetic Investigation of Communities by Reconstruction of Unobserved States) (Douglas et al., 2020). First, execute the normalize_by_copy_number.py script to normalize the ASVs. Second, the standardized ASVs table was entered into the predict_metagnomes.py script to generate the Kyoto Encyclopedia of Genes and Genomes (KEGG)-based prediction of microbial pathway abundance.

### 2.7. Statistical analysis

The Shapiro–Wilk test was used for normal distribution of ruminal fermentation parameters and alpha diversity index data of the two groups, followed by Student’s *t*-test for comparison of means. The Wilcoxon rank-sum test was used to compare the relative abundance of bacterial taxa between the two groups. All the data were analyzed using the SPSS Statistics 22 (IBM, Chicago, United States) software. The data were expressed as means and standard error of means (SEM). The treatment effects was significant at *p* < 0.05, and the trends was discussed at 0.05 ≤ *p* < 0.10.

## 3. Results

### 3.1. Rumen fermentation parameters

The ruminal pH and MCP production did not differ between CON and NCG treatments, but the ruminal NH_3_-N concentration in CON was significantly greater (*p* < 0.01) than that in NCG (Table 2). The concentration of total VFA tended (*p* = 0.09) to be greater in NCG than that of CON (Table 2). Greater (*p* = 0.03) molar proportion of acetate was observed in NCG than that in CON group, thus lead to a tendency (*p* = 0.07) of greater Acetate/Propionate ratio in CNG group; meanwhile, the molar proportion of butyric acid tended (*p* = 0.09) to be higher in CON than that in NCG (Table 2). Whereas, the molar proportion of the rest individual VFA were not different between CON and NCG treatments.

**Table 2:** Effects of NCG supplementation on ruminal fermentation and microbial crude protein of Holstein dairy cows in Tibet.

Items	Treatments^1^		SEM^2^	*p*-value
	CON	NCG		
pH	6.90	6.89	0.103	0.89
NH_3_-N, mg/dL	9.84^a^	6.09^b^	1.161	< 0.01
MCP^3^, mg/mL	1.38	1.49	0.154	0.49
Total VFA^4^, mmol/L	55.31	66.43	5.735	0.09
Acetic acid, mmol/100 mmol	58.65^b^	61.70^a^	1.320	0.03
Propionic acid, mmol/100 mmol	21.25	19.93	0.767	0.11
Acetic/Propionic	2.80	3.12	0.174	0.07
Butyric acid, mmol/100 mmol	15.54	14.17	0.767	0.09
Isobutyric acid, mmol/100 mmol	1.11	1.07	0.091	0.46
Valeric acid, mmol/100 mmol	1.25	1.20	0.124	0.70
Isovaleric acid, mmol/100 mmol	2.19	1.86	0.311	0.30

Values within a row with different superscripts differ at *p* < 0.05.

^1^CON, basal diet, without additive; NCG, basal diet + 20 g NCG/d.

^2^SEM, standard error of means.

^3^MCP, microbial crude protein.

^4^VFA, volatile fatty acid.

### 3.2. Rumen microbial richness and diversity

**Figure 1:**
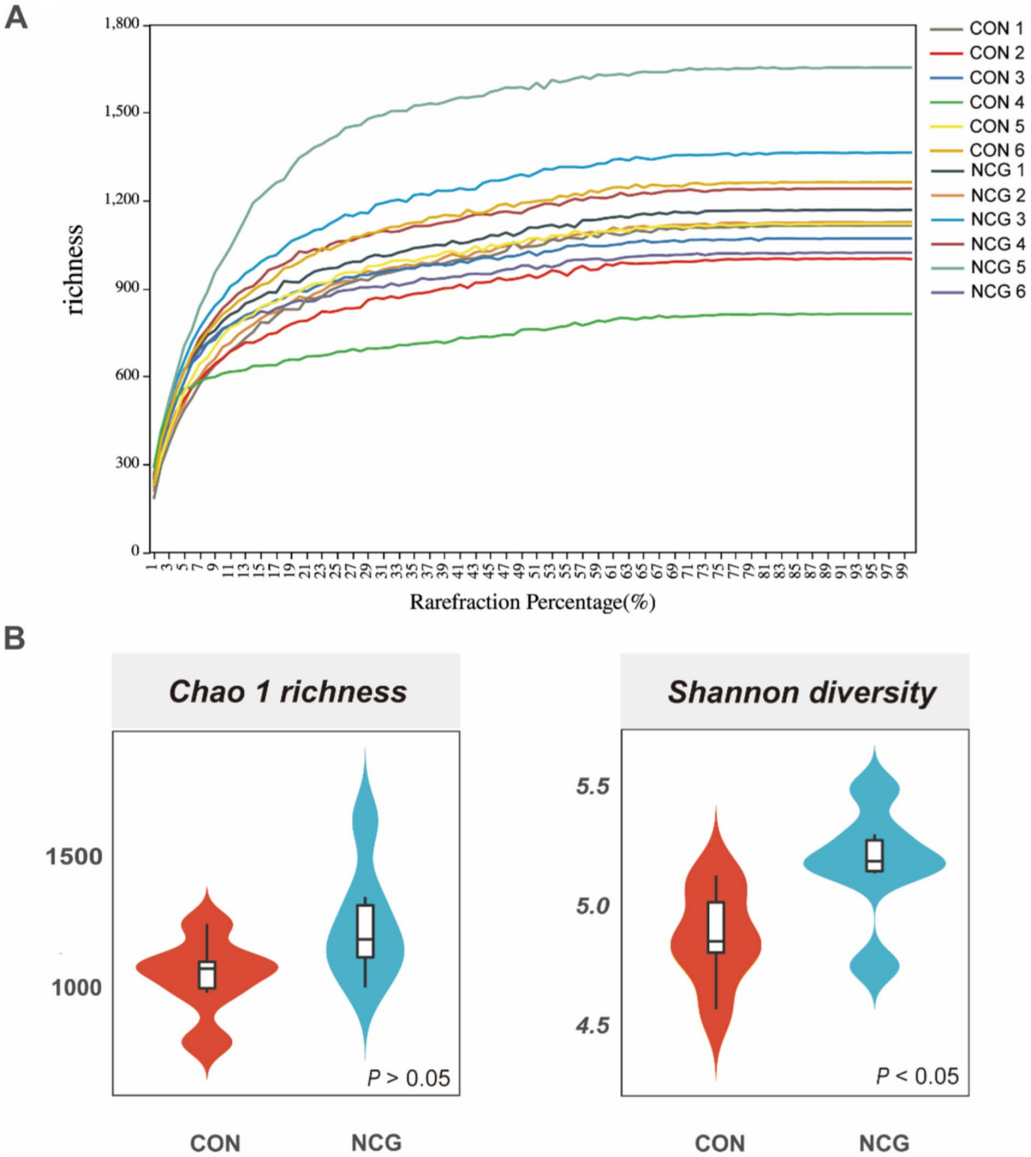
Rarefaction curve **(A)** and alpha diversity indices **(B)**; Chao 1 and Shannon indexes of ruminal bacteria of lactating cows fed CON and NCG diets. CON, basal diet; NCG, basal diet +20 g NCG/cow/d.

**Figure 2:**
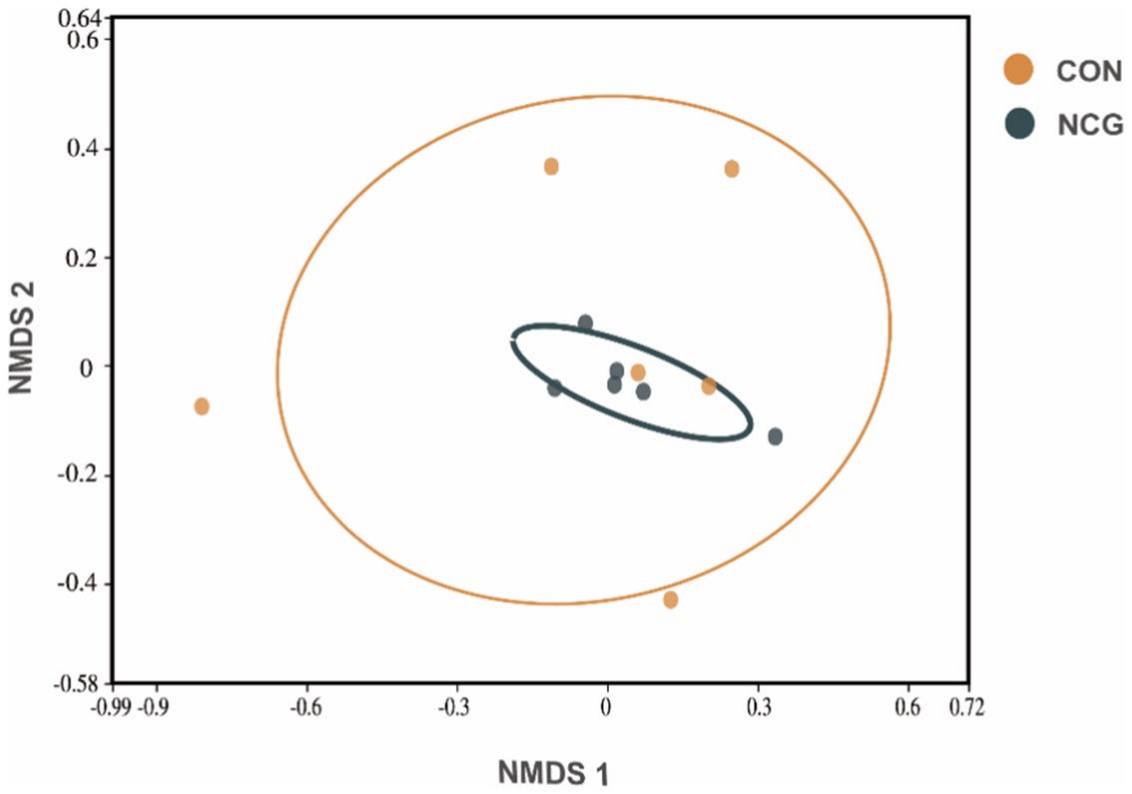
Non-metric Multidimensional Scaling (NMDS) analysis based on Bray Curtis (stress = 0.0994) distance of ruminal fluid samples of lactating cows fed diets with or without N-carbamoyl glutamate (NCG). The centroid of each ellipse represents the group mean, and the shape was defined by the covariance within each group. CON, basal diet; NCG, asal diet +20 g NCG/cow/d.

A total of 1,050,460 high-quality reads were obtained for all 12 rumen fluid samples, with an average of 87,538 ± 4,713 reads per sample (Supplementary Figure 1). Rarefaction curves showed that the sequencing depth covered essentially all species in all samples and accurately reflected microbial communities (Figure 1A). A total of 3,077 OTUs were obtained, with more than 95% annotated at phylum, class, and order level, approximately 80% annotated at family level, and only 56.87 and 31.04% annotated at genus and species level (Supplementary Table 1), respectively. Among which, 1839 were shared OTUs in the con and NCG cows, and 524 and 713 were unique OTUs in the con and NCG cows, respectively (Supplementary Figure 2). Alpha diversity indices including Chao 1 and Shannon diversity were analyzed, and the Chao 1 did not differ (*p* > 0.05) between treatments, while, the Shannon index was greater (*p* < 0.05) in NCG than that in CON (Figure 1B), suggesting that offering NCG to cows resulted in a greater ruminal bacteria diversity. The β-diversity was done by NMDS analysis, and the result showed that the ruminal bacteria community structure was greatly (*p* = 0.026) different between the CON and NCG groups (Figure 2; stress = 0.0994). As compared to the CON cows, the ruminal bacteria of cows received NCG supplementation had a clear cluster, indicating that they have a more similar microbial community structure.

### 3.3. Taxonomic analysis of ruminal microbial communities

Except some unclassified phyla, a total number of 19 phyla were identified in the ruminal microbiota of Holstein dairy cows in Tibetan plateau, with those greater than 1% in at least one group shown in Table 3. The five most abundant phyla were *Firmicutes* (averaged 54%), *Bacteroidetes* (averaged 17%), *Patescibacteria* (averaged 16%), *Proteobacteria* (averaged 6%), and *Tenericutes* (averaged 2%), accounting more than 95% of the entire bacteria identified. Notably, *Patescibacteria* was found to be a dominant phylum in Holstein dairy cow for the first time. At the family level, *Ruminococcaceae* (averaged 34%), *Saccharimonadaceae* (averaged 15%), *Lachnospiraceae* (averaged 11%), and *Prevotellaceae* (averaged 6%) were the four most abundant family (Table 4). Meanwhile, at the genus level, a total number of 77 genera were identified, and only the top 10 genera (including unassigned and uncultured) are listed in Table 5, with the *Candidatus_Saccharimonas* (averaged 15%), *Ruminococcus_1* (averaged 11%), *Papillibacter* (averaged 4%), *Ruminococcus_2* (averaged 4%), and *Lachnospiraceae_XPB1014_group* (averaged 4%) determined as the five most abundant genus. However, dietary NCG supplementation had no significant (*p* > 0.05) effect on relative abundances of ruminal bacterial at either phyla, family, or genus level.

**Table 3:** Relative abundances (%) of ruminal bacteria at phylum level in dairy cows received or not received NCG.

Phylum	Treatments^1^		SEM^2^	*p*-value
	CON	NCG		
*Firmicutes*	0.53	0.55	0.033	0.90
*Bacteroidetes*	0.18	0.15	0.026	0.91
*Patescibacteria*	0.15	0.17	0.032	0.89
*Proteobacteria*	0.07	0.05	0.013	0.91
*Tenericutes*	0.02	0.03	0.006	0.90
*Spirochaetes*	0.01	0.01	0.004	0.89
*Cyanobacteria*	<0.01	0.01	0.004	0.89
*Kiritimatiellaeota*	0.01	0.01	0.003	0.88
*Fibrobacteres*	<0.01	0.01	0.002	0.89
*Lentisphaerae*	0.01	<0.01	0.004	0.91

^1^CON, basal diet; NCG, control diet + 20 g NCG/cow/d.

^2^SEM, standard error of means.

**Table 4:** Relative abundances (%) of ruminal bacteria at family level in dairy cows received or not received NCG.

Family	Treatments^1^		SEM^2^	*p*-value
	CON	NCG		
*Ruminococcaceae*	0.34	0.35	0.037	0.89
*Saccharimonadaceae*	0.15	0.16	0.032	0.89
*Lachnospiraceae*	0.10	0.12	0.029	0.88
*Prevotellaceae*	0.07	0.05	0.018	0.99
*Bacteroidales_RF16_group*	0.04	0.05	0.009	0.88
*F082*	0.04	0.03	0.010	0.90
*Christensenellaceae*	0.03	0.04	0.006	0.88
*Clostridiales_vadinBB60_group*	0.03	0.02	0.014	0.89
(Unassigned)	0.04	0.06	0.009	0.88
Uncultured	0.05	0.05	0.012	0.91

^1^CON, basal diet; NCG, basal diet + 20 g NCG/cow/d.

^2^SEM, standard error of means.

**Table 5:** Relative abundances (%) of ruminal bacteria at genus level in dairy cows received or not received NCG.

Genus	Treatments^1^		SEM^2^	*p*-value
	CON	NCG		
*Candidatus_saccharimonas*	0.15	0.16	0.032	0.80
*Ruminococcus_1*	0.10	0.11	0.026	0.81
*Papillibacter*	0.04	0.05	0.011	0.80
*Ruminococcus_2*	0.05	0.04	0.023	0.80
*Lachnospiraceae_XPB1014_group*	0.05	0.04	0.012	0.86
*Prevotella_1*	0.04	0.03	0.008	0.87
*Christensenellaceae_R-7_group*	0.03	0.04	0.006	0.74
*Ruminococcaceae_UCG-010*	0.03	0.03	0.008	0.8
Unassigned	0.24	0.24	0.028	0.81
Uncultured	0.03	0.03	0.006	0.80

^1^CON, basal diet, without additive; NCG, basal diet + 20 g NCG/cow/d.

^2^SEM, standard error of means.

### 3.4. Correlation of rumen bacterial with fermentation parameters

**Figure 3:**
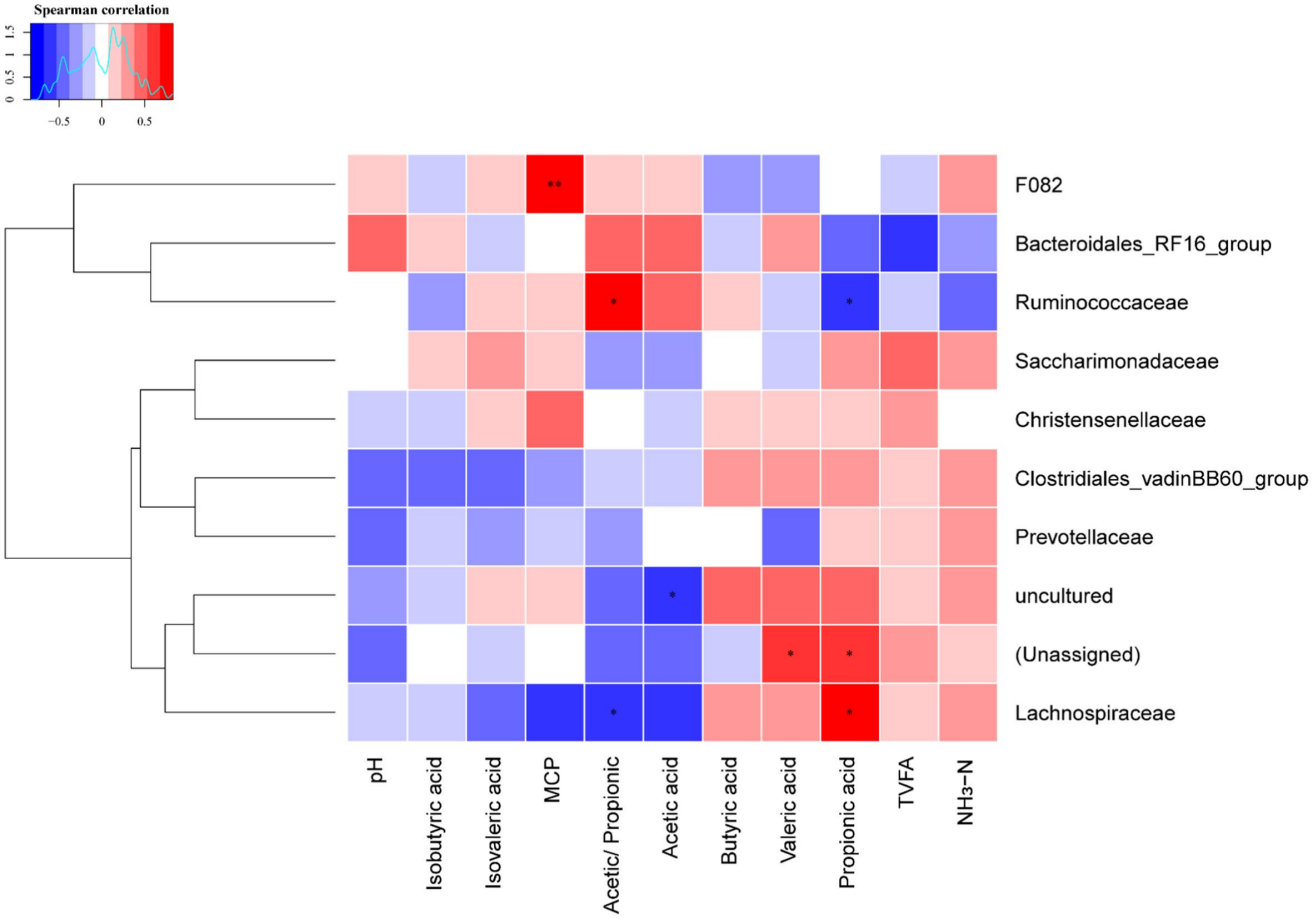
Heatmap of the correlations between the relative abundance of ruminal bacteria at family level and ruminal fermentation parameters. Blue color indicates negative correlations, and red color represents positive correlations. * Represents *p* < 0.05 and ** represent *p* < 0.01. MCP, microbial crude protein; TVFA, total volatile fatty acid; NH_3_-N, ammonia nitrogen.

The Spearman’s rank correlation analysis was used to investigate the correlations between ruminal fermentation parameters and rumen bacteria relative abundance at family level for dairy cows (Figure 3 and Supplementary Tables 2, 3). The production of ruminal MCP positively correlated with the relative abundance of family *F082* (*r* = 0.832, *p* = 0.001); the Acetic/Propionic positively (*r* = 0.713, *p* = 0.012) correlated with the relative abundance of family *Ruminococcaceae*, whereas it negatively (*r* = 0.832, *p* = 0.019) correlated with family *Lachnospiraceae*. In contrast, the molar proportion of propionic acid positively correlated with the relative abundance of family *Lachnospiraceae* (*r* = 0.699, *p* = 0.015), but negatively correlated with the family *Ruminococcaceae* (*r* = 0.678, *p* = 0.019). Attention should also be paid that the relative abundances of unassigned bacteria positively correlated with the molar proportion of propionic acid and valeric acid, as well as the uncultured bacteria negatively correlated with the molar proportion of acetic acid. This suggests that unassigned and uncultured bacteria are also played an important role in ruminal fermentation.

### 3.5. Rumen microbial function prediction

**Figure 4:**
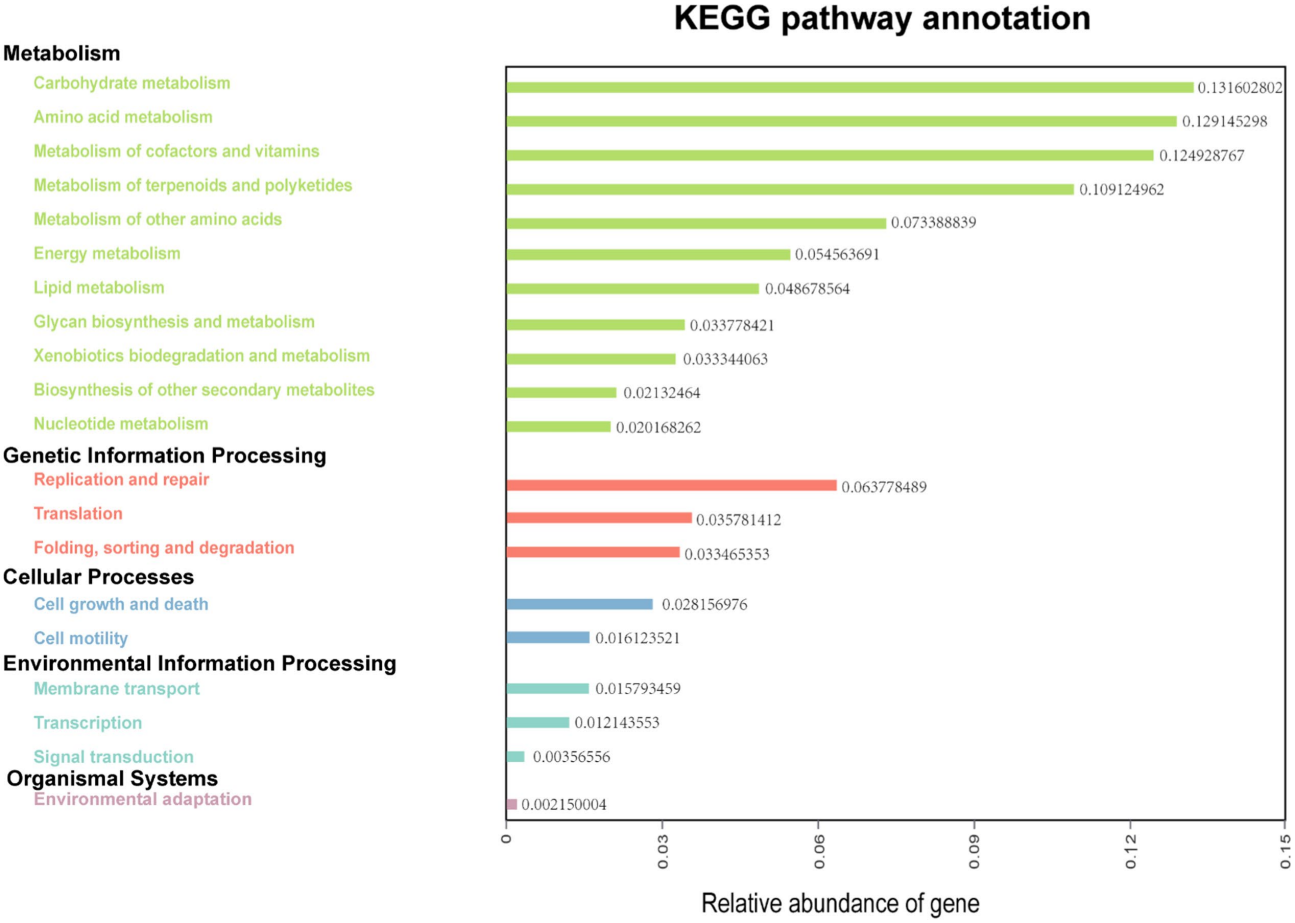
Predictive functional analysis showing relative abundance of assigned KEGG identity to the pathways of metabolisms at subsystem levels (KO_2_).

The functions of ruminal microbes were predicted using KEGG pathway analysis, with more than 77% of the bacteria of both treatments allocated to the function of “Metabolism” at level 1 of KEGG (Supplementary Figure 3). A total of 33 functional pathways were identified at the level 2 of KEGG orthologs (KO), with the top 20 pathways shown in Figure 4. In this potential pathway functional profile, the functional structure of the ruminal bacteria community identified in rumen fluid samples collected from dairy cows in Tibet was found to be dominated by pathways involved in metabolism (carbohydrates, amino acids, lipid, energy, and nucleotides), and genetic information processing (replication, and repair, translation). Moreover, the pathways responsible for cell growth and death, and cell motility were found to be dominant in pathways concerning cellular processes.

## 4. Discussion

Dietary supplementation of NCG altered ruminal fermentation, namely enhanced concentration of total VFA and acetic acid but lower NH_3_-N, which indicate enhanced ruminal N utilization. VFA can provide 75% of the energy for ruminants (Cheng et al., 2021), and also play an important role in immunity and growth of ruminants (Fan et al., 2020). The enhanced total VFA in NCG treatment was mainly because of increased molar proportion of acetic acid, suggesting a possibility increased fiber digestibility even though the nutrient digestibility were not determined. It was previously reported that NCG can promote the production of citrulline, which is further converted into fumaric acid and Arg under the action of enzymes (Hu et al., 2019). Fumaric acid is primary converted into acetic acid instead of lactic acid, which is beneficial for ruminal fermentation (Zhou et al., 2012). This might be the reason for the greater acetic acid production in cows fed NCG. Moreover, greater milk fat contents were determined in Jersey cows offered same dose of NCG like the current study (Liu H. et al., 2022; Liu Z. et al., 2022), suggesting that enhanced acetic acid are beneficial for milk fat syntheses. Dietary supplementation of NCG did not affect the molar proportion of propionate acid, thus lead to greater acetate to propionate ratio. Additionally, supplementation of NCG can promote urea cycle (Chacher et al., 2014), improve ruminal NH_3_-N utilization (Oba et al., 2005), and thus provide more N sources for MCP synthesis. It was proved in *in vitro* and *in vivo* studies that NCG can decrease ruminal NH_3_-N concentration (Chacher et al., 2013, 2014), enable rumen microbes to synthesize more MCP, thus reducing nitrogen excretion and improving nitrogen utilization (Chacher et al., 2014). The lowered ruminal NH_3_-N concentration in NCG cows was in consistence with previous reports by Chacher et al. (2013, 2014); however, only numerically greater contents of ruminal MCP were observed in NCG feeding cows. Taken together, supplementation of NCG to diet of Holstein dairy cows in Tibet do have benefits in improving ruminal fermentation and nutrient utilization, which will help the introduced animals to better adapt to the harsh environment of plateau hypoxia.

Biodiversity is essential for promoting the sustainability and productivity of numerous ecosystems. Similarly, the diversity of the rumen microbiota is closely related to metabolic ability, ruminal stability of ruminants, and health status. The current experiment studied the effects of diet add of NCG on rumen fermentation and rumen microbiota of Holstein dairy cows in Qinghai-Tibet Plateau. The results of 16 s rRNA gene sequencing indicated that dietary NCG supplementation resulted in an increased rumen microbial diversity of Tibetan Plateau dairy cows. We speculated that dairy cows of the NCG group would benefit from the increased microbial diversity, which will increase the stability of rumen environment and increase body energy to meet their survival requirements in the high-altitude and hypoxic environment in the Tibetan plateau. The more clustered ruminal bacteria community in NCG supports this speculation to some extends. Human studies have also shown that the diversity of intestinal microbiota improves the fermentation efficiency of dietary fiber and contributes to the stability of intestinal ecosystems (Tap et al., 2015). Moreover, the increased diversity of human gut microbes also reflects stronger metabolic capacity and improved health (Clarke et al., 2014).

The phyla *Firmicutes* and *Bacteroidetes* were dominant in the rumen of Tibetan Plateau dairy cows. *Firmicutes* and *Bacteroidetes* are consistently detected as the predominant phylum in rumen (Cui et al., 2019), suggesting that these bacteria play a significant role in rumen function and ecology of ruminants living in both low and high altitude. Furthermore, it should be noted that a high relative abundance of phyla *Patescibacteria* was detected in the rumen of Tibetan plateau dairy cows, which was not reported previously. Patescibacteria, also known as CPR, bacteria with special cell forms that are an important subset of the smallest known family of bacteria on Earth (He et al., 2015), it can be detected in wide range natural habitats (Rinke et al., 2013; Probst et al., 2018; Bokhari et al., 2020). Phyla *Patescibacteria* are commonly found in a variety of anaerobic environments such as wastewater treatment systems, soil, and human oral cavity, but are not a permanent member of the rumen environment (Wrighton et al., 2014). The plentiful *Patescibacteria* was not positive correlation with the nutrient index of water, indicating that the bacteria could adapt to oligotrophic environment (Herrmann et al., 2019). Cabello et al. (2019) were also able to assemble and obtain CPR MAGs in the bottom water of Belga Lake, again verified that CPR bacteria can live in an oligotrophic environment. Hence, we speculated that in high-altitude and low-oxygen regions, there may be some adaptive microbial community in the rumen microbes of Holstein cows. The enhanced microbes in the body may participate in important physiological functions of the body and maintain the health of the host, thus adapting to the harsh environment of high altitude and low oxygen. Nevertheless, their functional roles in Tibetan Plateau dairy cows still remain unclear and deserve further investigation.

Correlation analysis refers to the analysis of two or more variable elements with correlation, and is commonly applied to reveal the relationship between microbes and metabolites or fermentation parameters in rumen. In the current study, the relative abundances of *F082*, *Ruminococcaceae* and *Lachnospiraceae* family were found closely associated with ruminal fermentation and MCP production. The *Ruminococcaceae* and *Lachnospiraceae* belong to the *Firmicutes* phylum, was involved in the metabolism of a variety of carbohydrates which play a vital role in energy utilization (Lopes et al., 2021). The positive correlation between microbes and rumen VFA ensured the energy source of the host. Most of the VFAs formed in the rumen are absorbed across by the host ruminal epithelium (Zhang et al., 2016). Thus, dietary supplementation of NCG might possess the ability to more efficiently transport and absorb VFAs. The main VFA (acetic acid) in the rumen may cut down the abundance of *Escherichia coli* and maintain rumen health (Kong et al., 2014). This study showed that Acetic/propionic acid was positively correlated with *Ruminococcaceae*, and the response of VFA to rumen microbes indicated that dietary NCG supplementation could promote the interaction between microorganism and organism and adapt to the harsh growing conditions in Tibetan Plateau.

Rumen is a huge biological resource bank, and ruminal microbes play vital roles on the body’s immunity, nutrient absorption and degradation, and even enzyme metabolism. It is of great importance to excavate functional genes of rumen bacteria that are closely related to important nutritional physiological functions (Martin et al., 2010). In the current study, potential function prediction of ruminal bacteria of Tibetan Plateau dairy cows were carried out using KEGG pathway analysis. The results indicate that the most of ruminal bacteria were enriched in carbohydrate metabolism and amino acid metabolism at level 2 of KEGG pathways. This suggests that most ruminal bacteria are engaged in energy and amino acid metabolism so that to let the cows better adapt to the high-altitude hypoxic environment. Dietary NCG supplementation can increase endogenous arginine synthesis, promote amino acid metabolism, and increase amino acid utilization (Hu et al., 2019), which is identical with the results of functional prediction. In addition, genes related to replication and repair were the most abundant among genetic information processing. Studies have suggested that low oxygen and high ultraviolet radiation may cause DNA and protein damage in a plateau environment, while genes associated with replication and repair may help reduce damage to biomolecules (Fan et al., 2020). However, our results are based only on functional predictions of rumen bacteria. Further direct sequencing of the rumen metagenome of plateau cattle is needed to more accurately explore the role of these genes in NCG addition and environmental adaptation.

In conclusion, dietary NCG supplementation (20 g/d/head) reduced ruminal NH_3_-N concentration and enhanced ruminal total VFA production, especially acetic acid, suggesting enhanced N utilization and energy acquisition in rumen of Holstein dairy cows introduced to Tibet. The enhanced ruminal fermentation and N utilization is likely due to the enhanced ruminal microbial diversity, which needs to be further studied.

## Data availability statement

The datasets presented in this study can be found in online repositories. The names of the repository/repositories and accession number(s) can be found below: https://www.ncbi.nlm.nih.gov/search/all/?term=PRJNA900013.

## Ethics statement

The animals and experimental procedures used in this study were approved by the Institutional Animal Care and the Use Committee, Institute of Subtropical Agriculture, Chinese Academy of Sciences, Changsha, PR China (Approval number: ISA-2019-0115).

## Author contributions

CZ and DX were involved in the methodology and conceptualization. YW and AJ conducted the experiment. JZ and YW analyzed the sequencing data, interpreted the prepared the tables and figures, and wrote the manuscript. TR, CZ, DX, and JZ reviewed and edited the manuscript. CZ, BL, and ZT were involved in project administration. All authors have read and agreed to the published version of the manuscript.

## Funding

This work was jointly supported by the Special Item of Regional Collaborative Innovation in Tibet Autonomous Region and Science (No. QYXTZX-LS2021-01), the Science and Technology Service Network Plan of Chinese Academy of Sciences (No. KFJ-STS-QYZD-168), (No. XDA26040306) and the Earmarked Fund for China Agriculture Research System (No. CARS-37).

## Conflict of interest

The authors declare that the research was conducted in the absence of any commercial or financial relationships that could be construed as a potential conflict of interest.

The reviewer HN declared a shared affiliation with the authors JZ, YW, AJ, and CZ to the handling editor at the time of review.

## Publisher’s note

All claims expressed in this article are solely those of the authors and do not necessarily represent those of their affiliated organizations, or those of the publisher, the editors and the reviewers. Any product that may be evaluated in this article, or claim that may be made by its manufacturer, is not guaranteed or endorsed by the publisher.

## Footnotes

^1^ http://drive5.com/uparse/
