# Supplementary material for: Effects of dietary N-carbamylglutamate on rumen fermentation parameters, and bacterial community diversity of Holstein dairy cows in Tibet
Source: Front Microbiol. 2023 May 9;14:1101620. doi: 10.3389/fmicb.2023.1101620 (PMC10203405; doi:10.3389/fmicb.2023.1101620)
Supplement: Supplementary file 1 [file Data_Sheet_1.docx]

**Supplementary files**

**Supplementary Table 1.** Overview of operational taxonomic unit (OTU) annotation for all ruminal fluid samples

| **Item** | **Number or proportion** | **Dominant species** |
| --- | --- | --- |
| OTU catalogue^1^ | 3,077 | - |
| Kingdom level | 100% | - |
| Phylum level | 97.69% | *Firmicutes, Bacteroidete, Patescibacteria* |
| Class level | 97.21% | *Clostridia, Bacteroidia, Mollicutes* |
| Order level | 95.16% | *Clostridiales, Bacteroidales, Mollicutes_RF39* |
| Family level | 79.98% | *Ruminococcaceae, Saccharimonadaceae, Lachnospiraceae* |
| Genus level | 56.87% | *Candidatus_Saccharimonas, Ruminococcus_1, Ruminococcus_2* |
| Species level | 31.04% | *Dactylosporangium_sp._PENDO-1803,*  *uncultured_rumen_bacterium_4C0d-6*  *uncultured_bacterium* |

OTU = operational taxonomic unit.

^1^Total number of operational taxonomic units for all samples.

**Supplementary Table 2.** Correlations between the bacteria family abundance with rumen fermentation parameters.

| **Sampels** | **Ruminococcaceae** | **Saccharimonadaceae** | **Lachnospiraceae** | **Prevotellaceae** | **Unassigned** | **uncultured** | **Bacteroidales_RF16_group** | **F082** | **Christensenellaceae** | **Clostridiales_vadinBB60_group** | |
| --- | --- | --- | --- | --- | --- | --- | --- | --- | --- | --- | --- |
| TVFA | 0.81 | 0.08 | 0.73 | 0.66 | 0.31 | 0.54 | 0.05 | 0.68 | 0.39 | 0.65 |  |
| Acetic acid | 0.16 | 0.43 | 0.07 | 0.90 | 0.20 | 0.02 | 0.08 | 0.48 | 0.48 | 0.71 |  |
| Propionic acid | 0.01 | 0.37 | 0.01 | 0.61 | 0.02 | 0.16 | 0.11 | 0.83 | 0.80 | 0.43 |  |
| Acetic/ Propionic | 0.01 | 0.33 | 0.01 | 0.40 | 0.10 | 0.15 | 0.09 | 0.71 | 0.95 | 0.52 |  |
| Isobutyric acid | 0.35 | 0.57 | 0.71 | 0.48 | 0.83 | 0.78 | 0.49 | 0.69 | 0.73 | 0.11 |  |
| Butyric acid | 0.69 | 0.99 | 0.37 | 0.83 | 0.58 | 0.19 | 0.61 | 0.26 | 0.54 | 0.43 |  |
| Isovaleric acid | 0.65 | 0.44 | 0.15 | 0.35 | 0.81 | 0.68 | 0.57 | 0.749479 | 0.513513 | 0.16 |  |
| Valeric acid | 0.58 | 0.74 | 0.24 | 0.14 | 0.037 | 0.18 | 0.44 | 0.28 | 0.716184 | 0.45 |  |
| NH3-N | 0.12 | 0.29 | 0.39 | 0.43 | 0.66 | 0.37 | 0.25 | 0.28 | 0.93 | 0.28 |  |
| MCP | 0.63 | 0.74 | 0.06 | 0.81 | 0.85 | 0.57 | 0.97 | 0.00 | 0.10 | 0.41 |  |
| pH | 0.99 | 0.93 | 0.61 | 0.18 | 0.16 | 0.26 | 0.21 | 0.70 | 0.50 | 0.12 |  |

**Supplementary Table 3.** The correlations between the bacteria family abundance with rumen fermentation parameters.

| **Sampels** | **Ruminococcaceae** | **Saccharimonadaceae** | **Lachnospiraceae** | **Prevotellaceae** | **Unassigned** | **uncultured** | **Bacteroidales_RF16_group** | **F082** | **Christensenellaceae** | **Clostridiales_vadinBB60_group** | |
| --- | --- | --- | --- | --- | --- | --- | --- | --- | --- | --- | --- |
| TVFA | -0.07 | 0.52 | 0.11 | 0.13 | 0.31 | 0.19 | -0.57 | -0.13 | 0.27 | 0.14 |  |
| Acetic acid | 0.42 | -0.25 | -0.54 | -0.04 | -0.39 | -0.65 | 0.52 | 0.22 | -0.22 | -0.11 |  |
| Propionic acid | -0.67 | 0.27 | 0.69 | 0.16 | 0.66 | 0.42 | -0.48 | -0.06 | 0.08 | 0.25 |  |
| Acetic/ Propionic | 0.71 | -0.30 | -0.67 | -0.26 | -0.49 | -0.44 | 0.51 | 0.11 | -0.02 | -0.20 |  |
| Isobutyric acid | -0.29 | 0.18 | -0.11 | -0.22 | 0.06 | -0.09 | 0.21 | -0.12 | -0.11 | -0.48 |  |
| Butyric acid | 0.12 | -0.01 | 0.27 | 0.06 | -0.17 | 0.40 | -0.16 | -0.34 | 0.19 | 0.25 |  |
| Isovaleric acid | 0.14 | 0.24 | -0.44 | -0.29 | -0.07 | 0.13 | -0.18 | 0.10 | 0.20 | -0.42 |  |
| Valeric acid | -0.17 | -0.10 | 0.36 | -0.45 | 0.61 | 0.41 | 0.24 | -0.33 | 0.11 | 0.23 |  |
| NH3-N | -0.47 | 0.32 | 0.27 | 0.25 | 0.13 | 0.27 | -0.35 | 0.33 | 0.02 | 0.33 |  |
| MCP | 0.15 | 0.10 | -0.55 | -0.07 | -0.06 | 0.18 | -0.01 | 0.83 | 0.49 | -0.25 |  |
| pH | 0.00 | 0.02 | -0.16 | -0.40 | -0.43 | -0.35 | 0.38 | 0.12 | -0.21 | -0.46 |  |


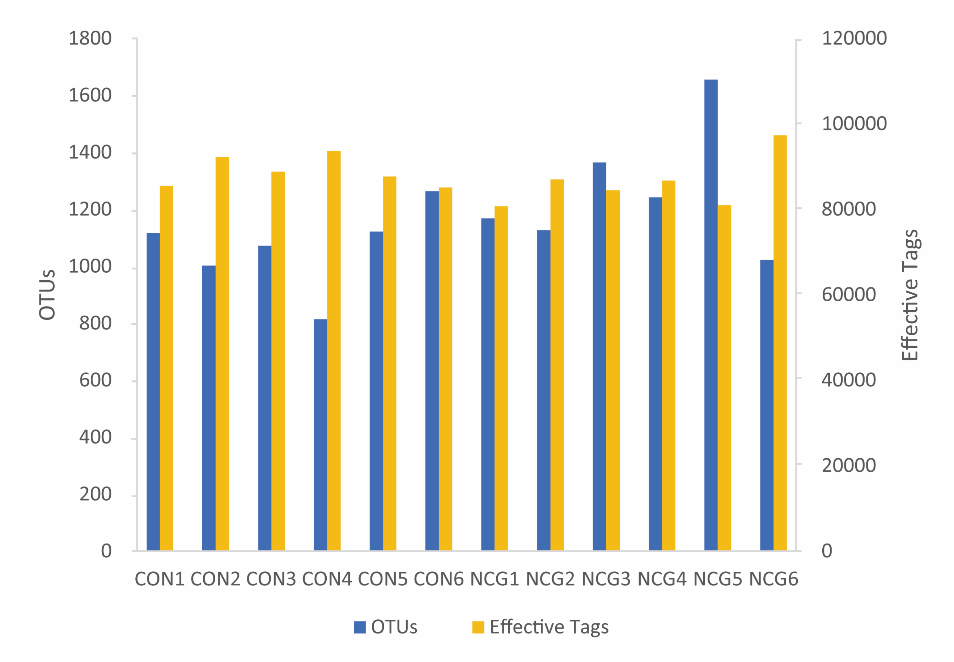


**Supplementary Figure 1.** Number of Tags and operational taxonomic units (OTU) acquired from the 16S rRNA gene sequencing in ruminal microbiota of lactating dairy cows fed CON or NCG diets.

CON = basal diet, without additive; NCG = basal diet + 20 g NCG/cow/d.


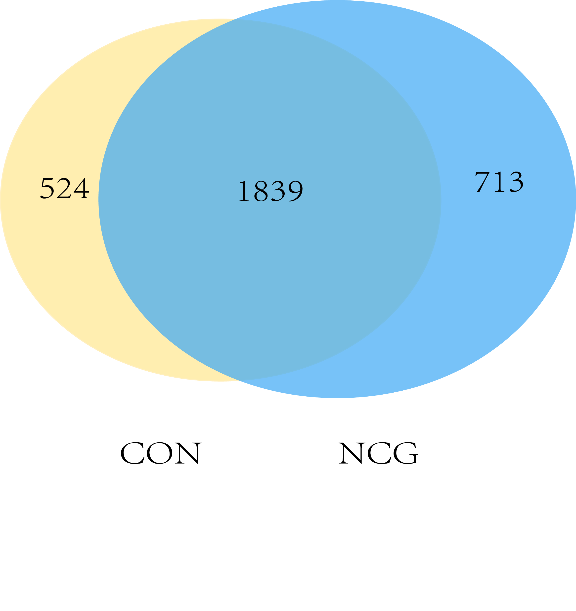


**Supplementary Figure 2.** Shared and special operational taxonomic units (OTU) of ruminal microbials of lactating dairy cows fed CON or NCG diets.

CON = basal diet, without additive; NCG = basal diet + 20 g NCG/cow/d.


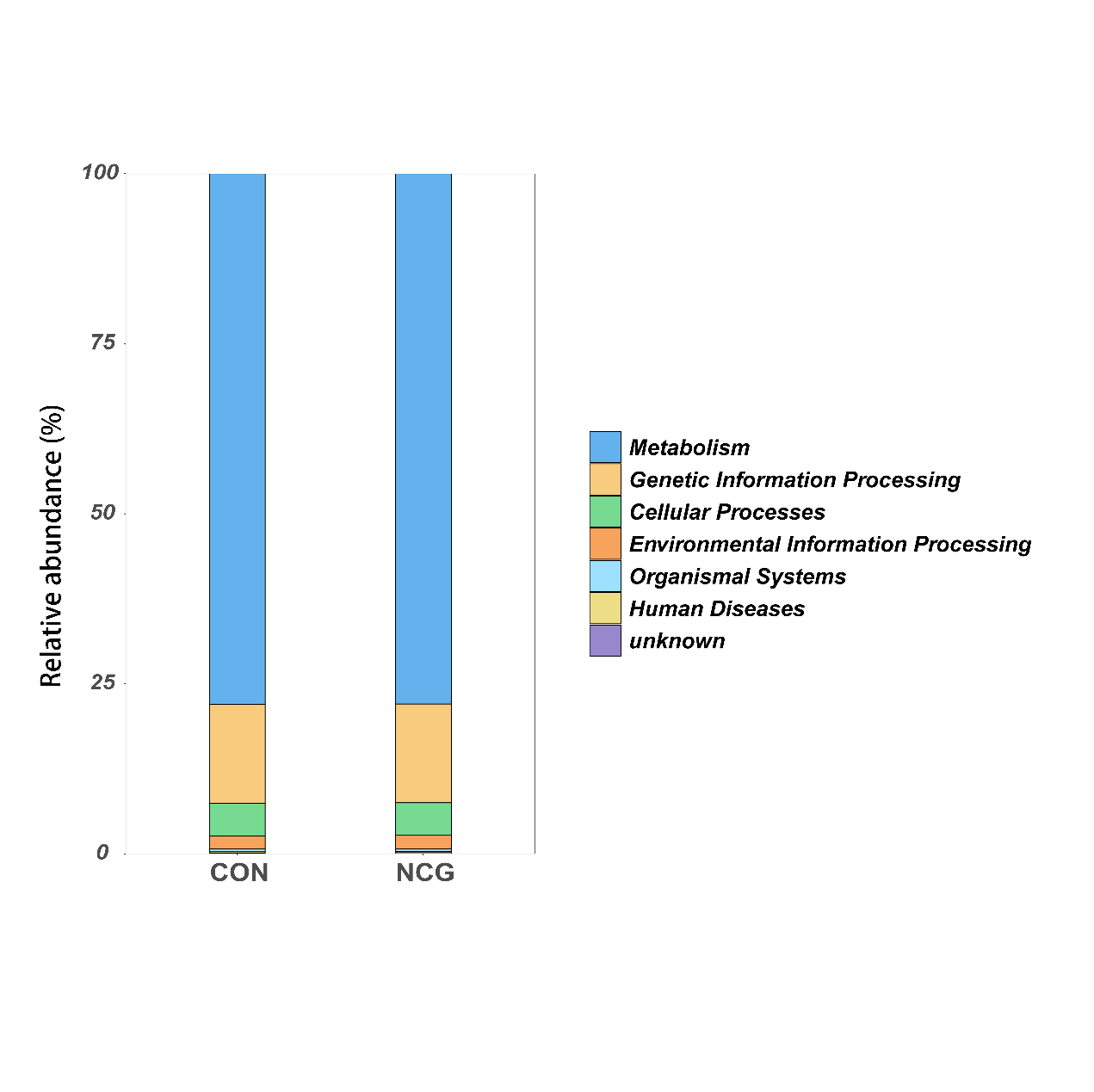


**Supplementary Figure 3.** Functional predictions for rumen microbiota with different KEGG pathways for two groups (CON and NCG), and KEGG pathways at Level 1 (*P* > 0.05).

CON = basal diet, without additive; NCG = basal diet + 20 g NCG/d/cow.
